# Human Milk—The Biofluid That Nourishes Infants from the First Day of Life

**DOI:** 10.3390/foods13091298

**Published:** 2024-04-24

**Authors:** Nikoleta Lugonja, Vesna Marinković, Mira Pucarević, Srdjan Miletić, Nataša Stojić, Dragan Crnković, Miroslav Vrvić

**Affiliations:** 1 Institute of Chemistry, Technology and Metallurgy, National Institute of the Republic of Serbia, University of Belgrade, Njegoševa 12, 11000 Belgrade, Serbia; srdjan.miletic@ihtm.bg.ac.rs; 2 Institute of Neonatology, Kralja Milutina 50, 11000 Belgrade, Serbia; vesnam555@gmail.com; 3 Faculty of Environmental Protection, Educons University, Vojvode Putnika 87, 21208 Sremska Kamenica, Serbia; mira.pucarevic@educons.edu.rs (M.P.); natasa.stojic@educons.edu.rs (N.S.); mmvrvic@bremgroup.com (M.V.); 4 City Public Health Institute of Belgrade, Blvd. Despot Stefana 54a, 11108 Belgrade, Serbia; dragan.crnkovic@zdravlje.org.rs

**Keywords:** human milk, infant feeding, milk quality, microplastic, milk bank

## Abstract

Human milk is a biofluid with a unique composition among mammalian milks. Besides this milk’s major components, its bioactive compounds, like hormones, immune factors, and oligosaccharides, are unique and important for infant growth and development. The best form of nutrition for term and preterm infants is the mother’s own milk. However, in the absence of the mother’s own milk, donor milk should be made available. Milk banks support neonatal intensive care units by providing preterm infants with human milk that generally has reasonable nutritive value for this sensitive population. However, neither mother’s own milk nor donor milk has sufficient energy content for the growth of preterm babies, so adequate human milk supplementation is crucial for their progress. Due to the different characteristics of human breast milk, as well as ubiquitous environmental pollutants, such as microplastics, new methods are required for monitoring the quality and characteristics of human milk, which will lay a solid foundation for the further development and progress of human milk research.

## 1. Introduction

Human milk is the best food for newborns within the first hour of life, and the only recommended source of nutrition for infants for the first six months, followed by complementary feeding for up to two years or beyond according to the World Health Organization (WHO) [1,2]. In addition to essential nutrients, human milk contains an array of bioactive components that promote growth and development and help maintain a healthy microbiota and immune system in infants. Based on its nutritional composition and biological activity, breast milk is considered the “gold standard” in the nutrition of newborns and infants [1,2,3,4]. Human milk contains nutritional factors, which, with their bioactive components, such as hormones, growth factors, enzymes, vitamins, and immune factors, contribute to this milk’s properties as a medicine for this sensitive group of infants. With its content of nutrients, especially human milk oligosaccharides, human milk stimulates the development of bifidogenic bacteria and microbiota in infants [5,6,7]. 

Due to its unique and natural composition, human milk is ideally suited to support crucial developmental processes in infancy. There are numerous health benefits associated with breastfeeding and human milk, both for mothers (facilitator of mother–infant bonding, reduced risks of breast and ovarian cancer, hypertension, and type 2 diabetes) [8,9,10,11,12] and their infants (both short-term and long-term) [13,14,15,16,17,18,19]. For infants, short-term benefits include lower incidences of diarrhea, pneumonia, atopic dermatitis, and sudden infant death syndrome, while long-term benefits include reduced risks of type 2 diabetes, leukemia, autism spectrum disorder, and obesity. Breastfeeding also has a positive impact on the child’s intelligence quotient and social behavior [13,14,15,16,17,18,19]. 

The main goal of this paper is to highlight and emphasize the well-known and very complex meaning and importance of breast milk for the growth and development of the newborn, especially due to the presence of biologically active components unique only to breast milk and in particular, hormones, hormone-like substances, immune factors, and perhaps more importantly, human milk oligosaccharides (HMOs) as inherent prebiotics found only in this biofluid. We focus particularly on the influence that human milk has on the infant microbiome, the composition of which has been known for many years to have an almost decisive influence on a healthy and successful beginning of life. The second focus of this work refers to the importance of feeding preterm babies with human breast milk, to which we pay special attention in the context of considering human milk banks. Consideration of human milk is complicated by its special importance in the nutrition and development of preterm babies. Unfortunately, the global problem of microplastic pollution has not bypassed breast milk and breastfeeding [20,21]. Microplastics are certainly not the only pollutants to be found in human milk. However, they are massive and global pollutants. Therefore, the third major focus of this report is microplastics, which can be ingested by newborns either directly from the mother’s milk or indirectly through plastic pacifiers and nipples, bottles, or the mother’s skin. Through the main goal and these individual themes, an attempt is made to show the complexity and connection of newborns’ nutrition and healthy development with the nutritional and biological value of human milk while also pointing out the advantages for preterm infants of donor milk if the mother’s own milk is not used.

## 2. Nutritional and Biological Values of Human Milk

During the lactation period, the composition of milk varies among individuals and matures and changes to adapt to the needs of the newborn [22,23]. The key components of human milk include proteins (0.8–1%), lipids (3–5%), carbohydrates (6.9–7.2%), minerals and vitamins (0.2%), and water (87%) [24,25,26,27,28]. In addition to these components, human milk also contains immune factors, vitamins, hormones, growth factors, and prebiotic, and probiotic compounds, which stimulate the infant’s growth and the development of the infant’s microbiome. Various biological, ecological, hormonal, immunological, and social/behavioral factors synergistically influence the biological process of synthesis and secretion of human milk, affecting composition and lactation. Factors that regulate the development of the mammary gland are the anatomy of the breast, diet, and the hormonal milieu [29,30]. Circadian biology influences the secretion of milk, and particularly, the concentrations of fats, cholesterol, iron, melatonin, cortisol, and tryptophan have circadian patterns [22,23].

The composition of breast milk changes as the infant grows to meet their evolving nutritional needs. Colostrum is the first milk produced by a mother after giving birth. It contains higher contents of protein, immunoglobulins, hormones, enzymes, vitamins, and minerals, compared to mature milk [31,32,33,34,35]. At this early stage, the newborn’s gastrointestinal system is anatomically correct but functionally immature. The newborn’s functions of digestion and absorption of nutrients mature gradually during the first few months of life and depend on the activity of specific enzymes. Numerous bioactive substances present in breast milk provide protection against infections and contribute to immune system maturation, organ development, and healthy microbial colonization [36,37]. The wide range of breast milk’s bioactivity arises from the functionality of bioactive proteins and the gastrointestinal release of bioactive peptides derived from them. These peptides exhibit a spectrum of activities, including enhancing mineral absorption, modulating the immune response, exerting opioid-like effects, eliciting antihypertensive actions, and providing antimicrobial properties. Importantly, certain activities can be solely attributed to the released bioactive peptides, distinct from the parent proteins [38,39]. 

### 2.1. The Influence of Human Milk on Preterm Infant Development and Nutrition

Every year, 1 out of 10 newborns are born prematurely, before the 37th week of gestation, and most countries have experienced an increase in the rate of premature births over the last 20 years [40,41,42,43]. In Canada, 8% of newborns are born prematurely each year, in England and Wales 7%, in South Asia around 13%, but in some countries, the preterm birth rate exceeds 15%. Due to its population size, the preterm birth rate in India, at 13%, accounts for almost a quarter of the global number of preterm births. The mean preterm birth rate in Europe is 10% [44,45]. The United States recently experienced a 0.4% increase in the preterm birth rate in just one year, rising from 10.1% in 2020 to 10.5% in 2021 [46].

Ensuring proper nutrition for preterm infants is crucial, and human milk plays a vital role in meeting their nutritional needs. The nutritional needs of preterm infants significantly differ from those born at term. Premature infants have an underdeveloped gastrointestinal tract at birth, making them more susceptible to infections [43,47,48]. The colostrum from mothers of premature infants has a significantly higher protein content compared to mature milk and milk from mothers of term infants. Transitional milk from mothers of premature infants appears between 5 and 20 days into lactation. Transitional milk differs from colostrum in its consistency, being thinner, more watery, and often sweeter in taste. Transitional milk is individually less variable in its protein composition and antioxidant properties. The whey fraction of transitional milk contains the most vitamin C, the main antioxidant, and could be the safest fraction of human milk for long-term storage and fortification, making it suitable for donation to human milk banks [49,50,51,52,53,54,55,56].

Preterm babies, particularly susceptible to oxidative stress, have heightened vulnerability while they are required to be in neonatal intensive care units (NICUs) [57,58]. Fresh human milk possesses a distinct antioxidant profile, capable of inhibiting superoxide, hydroxyl, and peroxide radicals. Antioxidants, biologically active compounds integral to cellular processes, play a vital role in protecting cells from the deleterious effects of free radicals. The concentrations of antioxidants in human milk are specific and dependent upon various factors, including age, lifestyle, maternal dietary patterns, and the gestational age at birth. Antioxidants are an important factor in determining the quality of milk used for feeding premature infants, as these compounds contribute to the nutritional value of breast milk and provide protection against oxidative stress. There are no significant differences in the redox properties of term and preterm milk. Both lipophilic antioxidants (such as tocopherols, retinol, and carotenoids) and hydrophilic antioxidants (such as ascorbic acid, polyphenols, low molecular weight thiols, casein, whey proteins, and antioxidant enzymes) collaborate synergistically within breast milk to furnish antioxidant defense to infants and neutralize free radicals. This defense system includes radical scavengers (vitamins E and C), transition metal chelators, and enzyme systems, like copper–zinc SOD, mitochondrial SOD, glutathione peroxidase (GP), and glutathione reductase (GR) [59,60,61]. However, the antioxidant activity of enzymes differs across different lactation stages: colostrum and mature milk. Supplementation with antioxidants holds promise in averting disease development, modulating neonatal immune function, and enhancing overall vitality. The combined antioxidant activities of these molecules have recently been defined as the total antioxidant capacity (TAC) [59,60,61].

Human milk has direct pharmacological relaxation effects on non-vascular smooth muscle, which is crucial for muscle relaxation in the infant stomach. Conversely, infant formulas demonstrate negligible impact on smooth muscle contractions, with some formulas even increasing contraction frequency. This can be attributed, in part, to higher levels in human milk than in infant formula of superoxide dismutase (SOD), an enzyme known for its capacity to induce relaxing effects in addition to its anti-inflammatory properties [62]. Angiotensin-converting enzyme, derived from casein, along with the inhibitory tripeptides, isoleucine-proline-proline and valine-proline-proline, present in breast milk, exerts anti-hypertensive effects in humans and alleviates the development of hypertension within experimental models. Research indicates the potential of peptides derived from human milk as therapeutic agents against hypertension, due to their ACE-inhibitory activity and ability to permeate Caco-2 monolayer cells [63,64].

The gut’s innate immune system is critical for the development and health of newborns in early life. The gut immune systems of preterm and term infants differ at birth, and this altered microbiome can lead to the long-term risk of immune dysfunction in preterm babies. Insufficiency of intestinal innate immune cells is a significant symptom of gut immune system immaturity in preterm babies. Lower villi height and a thinner muscle layer in the intestine have been observed in multiple studies, suggesting inadequate function of the gut mechanical barrier in preterm babies [38]. Additionally, aside from the deficiency in the number of gut immune cells, innate immune cells in the gut can also be dysfunctional in preterm babies. Secretion deficiency and impaired mucosal restitution are also insufficient, largely due to low expression of trefoil factor peptides. Over recent decades, evidence has emerged indicating the existence of a bidirectional communication axis involving the gut–brain–microbiota, defined as the gut–brain–microbiota axis. In this context, given the limited data showing a correlation between microbiota dysbiosis and neurodevelopmental disorders in premature infants, there is growing interest in better understanding the impact of the gut–brain–microbiota axis on clinical outcomes for preterm babies and how this understanding can lead to alternative preventive, diagnostic, and therapeutic strategies, such as probiotic supplementation of the premature in NICU [65].

### 2.2. The Use of Donor Milk for Preterm Nutrition

The supply of breast milk is often limited for preterm babies, so they are unable to rapidly receive from breast milk healthy doses of maternal antibodies and bacteria or adequate amounts of nutrients. To ensure an adequate supply of breast milk, human milk banks allow preterm infants whose mothers are unable to provide enough to receive donor human milk. Donor milk is a preferable alternative to feeding preterm infants with formula when the mother’s own milk is unable to be provided. Donor milk provides the nutritional and immunological benefits of breast milk and decreases rates of necrotizing colitis, late-onset sepsis, and retinopathy of prematurity in infants, but also results in lower levels of metabolic syndrome, blood pressure, and insulin and leptin resistance at adolescence, compared to premature infants on infant formula [66,67]. However, compared with mother’s own milk, donor milk has different effects due to the changes that occur after thermal treatment during meal preparation. Total lipids and proteins decrease after storage and pasteurization, as does albumin, which does not differ from that in preterm milk but decreases with pasteurization [68]. Premature infants may not receive the necessary amount of macronutrients from preterm breast milk due to variations in the individual components of the milk. In fact, a diet of preterm breast milk alone over an extended period can lead to nutritional deficits, compared to infants who are fed milk supplemented with fortifier or infant formula designed for premature babies. Milk fortifier, used to supplement human milk, has been developed to support breastfeeding in premature infants’ diets by increasing the proteins, vitamins, minerals, and energy. As the composition of human milk can be altered by storage and pasteurization, these factors need to be considered for milk management by milk banks. Nutritional management of high-risk infants using human milk requires personalized, adaptive, or targeted strategies for fortification based on milk composition measurement and growth monitoring [69,70].

The largest quantity of donated milk comes from mothers of preterm infants, due to the mismatch between the capacity of premature babies to directly consume it and the amount of milk produced. Proper brain development requires specific nutrients, such as docosahexaenoic acid and choline. Preterm infants have lower levels of choline but comparable levels of arachidonic acid and docosahexaenoic acid to term infants. Supplementing preterm human milk with a combination of choline, arachidonic acid, and docosahexaenoic acid can improve the nutritional status of preterm infants and support their healthy development [49,71,72,73]. 

For preterm infants, the availability of human milk in milk banks is crucial [74,75,76]. The practice of human milk donation has a long history, dating back to a time when children were nursed by friends, relatives, or even strangers. Premature infants benefit from human milk as it protects them from medical conditions like necrotizing enterocolitis and sepsis. Established human milk banks collect, screen, store, process, and provide high-quality and biologically safe donor milk, according to local and national guidelines and recommendations. Donating women are carefully selected, screened, and serologically tested to exclude infectious diseases (HIV, Hepatitis B, Hepatitis C, and other bacteria and viruses according to local concerns), use of drugs, medications, and cigarettes, and recent injury, blood transfusion, tattoo, piercing, or needle stick injury. Donor milk can be microbiologically tested before and after pasteurization, depending on local and national guidelines. 

Heating donor milk by pasteurization is a common thermal treatment to reduce the number of microorganisms the food contains. Holder pasteurization, i.e., heating milk at 62.5 ± 0.5 °C for 30 min followed by rapid cooling before transfer to the freezer and storage, eliminates some bacteria but changes the functions and contents of proteins, lipids, and heat-labile bioactive components in the milk. Pasteurized donor milk has lower lactoferrin, insulin, and prolactin concentrations compared to mother’s own milk and lower levels of other bioactive components (secretory IgA, lysozyme, vitamins, enzymes, cytokines, and antioxidant activity) [38,39]. Pasteurized donor milk has dramatically less leptin but more cortisol than non-pasteurized milk. Although heat treatment diminishes some properties of milk, pasteurized donor milk is still highly preferable to formula [77]. 

Human milk banks are the most important providers of donor milk, and although they do not reduce breastfeeding rates at discharge, they do lower formula use in the first weeks. Milk banks around the world are organized differently in various countries, and in many countries, milk donation to milk banks is limited by religious, political, or health factors [75,76,78,79]. Brazil has the most extensive and perhaps best-organized system, with 222 human milk banks and 217 collection centers, which can serve as an exemplary lesson for other countries. There are 282 active milk banks in Europe, and 18 more are planned, all coming under the same regulation for milk donation, following the guidelines of the European Milk Bank Association (EMBA). Italy and Germany have the largest number of human milk banks. In 2020, there were 756 milk banks in 66 countries. The number of milk banks is increasing in low- and middle-income countries. In the region of South-East Europe, there are only nine milk banks in five countries. Greece has four active milk banks, Serbia has two active and one planned milk bank, while Croatia, Bulgaria, and Romania each have one milk bank [76,78,80,81]. 

### 2.3. The Relevance of Milk Type on Biologically Active Components

Each type of human milk (preterm, term) has immunomodulating bio-factors that protect the intestine against injuries due to oxidative stress and inflammation and prevent pathogenic translocation into the bloodstream. Immunomodulating bio-factors (lactoferrin, lysozyme, cytokines, secretory IgA, IgG, and IgM) are produced by different immune cells, decrease over time, and become stable when breast milk matures [82,83]. Lactoferrin, secretory IgA, and lysozyme comprise the important compounds in the whey fraction of human milk. Two protective proteins, secretory IgA and lactoferrin, comprise 10% of milk weight for the first 48 h after birth. Lactoferrin has antimicrobial activity and can directly or indirectly protect neonates against infections caused by bacteria. The concentration of lactoferrin in preterm milk is higher than in term milk. However, preterm infants receive small amounts of colostrum compared to term infants. Enteral supplementation of lactoferrin contributes to the prevention of sepsis and necrotizing enterocolitis in preterm babies by modifying the gut microbiome and enhancing systemic immunity. The utilization of lactoferrin by beneficial commensal bacteria underscores its role in activating gut immune signaling pathways within the intestinal immune system [38,39]. Secretory IgA is synthesized by the mother’s plasma cells against specific antigens, which is carried out through gut-associated lymphoid tissue and bronchus-associated lymphoid tissue when the mother is exposed to the respiratory or gastrointestinal tract to antigens. Ingestion of milk provides to infants passive secretory IgA antibodies against antigens, and these antibodies prevent bacterial attachment to enteric tissues through steric hindrance. Lactoferrin synergistically acting with secretory IgA absorbs enteric iron and prevents the entry of pathogenic organisms. Mothers of infants with systemic infection and poor suckling have higher IgA levels in their breast milk [84]. 

The wide array of hormones in human milk includes leptin, adiponectin, ghrelin, insulin, resistin, obestatin, and apelin. Their role is in the regulation of infant development and health outcomes, contributing to the body composition and appetite control, regulating infant food intake, weight, and adiposity and preventing obesity and diabetes [34,85]. The concentration of leptin, insulin, obestatin, and resistin in human milk decreases throughout the lactation period, while glucocorticoids increase. Leptin in preterm milk is present in higher concentrations, compared to term milk, and is nearly threefold higher compared to donor milk. Since leptin decreases during lactation, and preterm infants have reduced leptin production, they are at high risk of leptin deficiency [5,86]. Ghrelin has a positive correlation between the 24 h milk intake and infant weight gain [87]. Obestatin is considered an antagonist to ghrelin, and its concentration in human milk is higher than in maternal serum, indicating synthesis in the mammary gland is an important factor for infant nutrition. There is no effect of gestational age on breast milk insulin levels, while preterm milk cortisol levels are lower compared to those of term milk. Preterm milk contains more prolactin than term milk, while there is no difference in follicle stimulating hormone and luteinizing hormone levels in the two milk types [77].

### 2.4. The Influence of Breast Milk on Infant Gut Microbiome

Another extremely important effect of human milk is its stimulatory impact on the growth of newborns’ microbiome. The first available and best natural prebiotics are HMOs. These, together with other milk macronutrients, stimulate the development of the infant gut microbiome from the first day of the infant’s life. There are over 160 different structures and isomers of HMO in human milk, and about 34 of these HMOs are not found in any other mammals [88,89,90]. Breastfeeding women synthesize different sets of oligosaccharides [91]. Variations in the composition of HMO depend on the genetics of the mother, that is, the secretory status. The number and profile of HMOs is defined by the expression of secretory and Lewis genes for certain glycosyl-transferases, especially fucosyl-transferases [92,93]. Secretory milk contains large amounts of α1-2 fucosylated oligosaccharides, while non-secretory milk has no α1-2 fucosylated oligosaccharides and lower concentrations of total oligosaccharides [94,95].

The amount of HMO in breast milk depends on the stage of lactation. The HMO content of colostrum is about 20.9 g/L, while in mature milk, it is 12.9 g/L [96]. Fucosylated and sialylated HMOs, in addition to their direct prebiotic effects, can also have an indirect competitive effect on the composition of the microbiome in the intestines, by preventing pathogen adhesion to the intestinal epithelium [37]. HMOs are non-digestible and structurally similar to mucosal glycans, acting on glycan-mediated processes, such as the earliest colonization of the gastrointestinal tract microbiome, immune system development, and pathogen infectivity [97]. HMOs have a role in shaping the microbiome of babies. Breast milk containing more secretor HMOs stimulates a higher abundance of *Bifidobacterium* in infants’ microbiomes compared to the levels in infants fed non-secretory milk, and in whom later colonization and the presence of more *Clostridium* spp. and *Enterobacteriaceae* in feces is measured. The milk of non-secretors does not contain 2′-Fucosyllactose (2′-FL) or other α-(1,2) fucosylated HMOs, or they are present in minimal amounts (weak secretors are found in some Asian populations) [90,96].

The types of bacteria that make up the gut microbiota of infants can affect their health. In exclusively breastfed infants, bifidobacteria (*Bifidobacterium infantis*, *Bifidobacterium breve*, *Bifidobacterium longum*) dominate, while the numbers of enterobacteria, *Escherichia coli*, and *Bacteroides* are low. The microbiota of infants fed infant formula is more diverse, with bifidobacteria as the dominant species (*Bifidobacterium adolescentis*, *Bifidobacterium catenulatum*), but also significant amounts of *Bacteroides*, *Clostridia*, and *Streptococcus*, due to the alkaline environment and the absence of prebiotic factors present in breast milk [92,93]. The species *Bifidobacterium longum* subsp. *infantis* contains enzymes that efficiently degrade all HMOs, while other bifidobacteria present in the infant gut microbiota do not contain enough enzymes for efficient use of all HMOs [98,99]. 

HMOs enhance the gastrointestinal barrier and promote a gut microbiome rich in *Bifidobacterium*, which helps protect against infection, strengthens the epithelial barrier, and generates immunomodulatory metabolites [100]. HMOs have various physiological functions, including potential support for the immune system, brain development, and cognitive functions [13,101]. The main role of HMOs in premature infants is to stimulate the production of a gut microbiota dominated by *Bifidobacterium*. Oligosaccharides and fructose in breast milk play a role in infant growth and body composition. There are intriguing associations of these two carbohydrate fractions with infant cognitive development, as research suggests that earlier exposure to oligosaccharide 2′-FL through breast milk could augment infant cognitive development during a critical window of brain development [101,102]. 

Most studies on gut microbiome development have focused on term infants, but health outcomes are also critical for preterm infants. The systemic physiological immaturity of very premature infants (born before 32 weeks of gestation) leads to numerous microbiome–organ interactions, the mechanisms of which are yet to be fully understood [103,104]. The gut microbiome is widely recognized as a critical factor in stimulating the development and function of the intestinal immune system after birth. However, the gut microbiome of preterm babies is at high risk of dysbiosis, which is strongly linked to harmful effects on immune system development and education in early life. Postnatal changes in microbiota composition can impact further development, morbidity, and long-term outcomes [65]. Disturbed and immature gut microbiota is often detected in preterm newborns and is linked to various factors, such as the increased susceptibility to inflammation in mothers of preterm babies, lack of mother’s own milk, rapid labor or cesarean section, excessive use of antibiotics during and/or after delivery, and the postnatal caregiving environment [103]. Bacteria, like *Bifidobacterium longum* subsp. *infantis*, can actually be underrepresented in the intestines in the modern conditions of raising children. Studies into the influence on the infant microbiome of diet composition are important, because dietary interventions offer the possibility of changing the microbiota in order to improve health [105,106,107].

## 3. Breastfeeding Rates of Term and Preterm Infants

Despite the clear benefits of natural infant feeding with breast milk, breastfeeding rates worldwide remain unsatisfactory. Globally, the breastfeeding rate is lower than the recommended level for ensuring the health of both mothers and infants. In the period from 2016 to 2022, according to WHO and UNICEF data, just 46% of newborns were initially breastfed within the first hour after birth, while collective guidelines aim for this rate to reach 70% by 2030 [108]. On a global scale, only 48% of infants are breastfed exclusively under six months of life, and 71% of those women breastfeed their children for at least one year. The percentage of children breastfed exclusively up to two years decreases to 45% [16,108,109]. In European countries, exclusive breastfeeding rates decline after four months, with breastfeeding rates ranging from 38% to 71% at six months [110,111]. Due to the WHO recommendation to introduce solid foods to babies aged between 4 and 6 months, the number of exclusively breastfed infants drops to 7% [112]. In the countries of South-East Europe, the percentage of breastfed infants is significantly lower than in other European countries [3]. Due to cultural, traditional, and historical differences, there is a lack of support for mothers, which is reflected in the low rates of breastfeeding and the mothers’ socio-economic status. This region needs milk banks to support mothers of preterm infants, but unfortunately, there is a lack of relevant assistance, which contributes to low breastfeeding rates. The highest breastfeeding rate is in Albania at 36.5%, while the lowest is in Croatia at 10.7%. The percentage of exclusively breastfed infants in Serbia is quite low, with only about one in four newborns being exclusively breastfed up to five months of age. This rate is even lower among newborns from vulnerable groups [109]. 

Mothers and their premature infants often face many obstacles to breastfeeding. Premature babies are at risk of feeding difficulties due to the immaturity of their neurological and motor systems, which are further exacerbated in those with underlying complications. While the ultimate nutritional goal is to achieve complete enteral feeding through breastfeeding in preterm babies, many variables influence the mother–infant relationship and the establishment and continuation of lactation, including nutritional, biological, psychological, cultural, and social components, all of which differ significantly in preterm births compared to term births [113,114,115,116,117]. Preterm birth is associated with a higher risk of short-term complications than term birth, making early minimal enteral feeding with human milk, especially colostrum, extremely important [118,119,120]. However, there is limited evidence about current breastfeeding practices for premature infants. According to a study conducted in China, 66% of preterm babies are breastfed at NICU discharge, while only 22.5% of all babies are exclusively breastfed during the first 6 months. In Malaysia, over 80% of babies are breastfed at NICU discharge, and in Italy, 60.4%. US data showed that making human milk available from a human milk bank increased breast milk feeding at NICU discharge by 10% [121,122,123]. A cohort study carried out in Denmark involving 1221 mothers and their 1488 babies born at gestational ages of 24 to 36 weeks showed that 99% of mothers initiated breastfeeding, and 68% of babies were exclusively breastfed upon NICU discharge. Nearly all (99%) Danish mothers of premature babies who had planned to breastfeed actually initiated breastfeeding in the NICU, and 79% of preterm newborns had their first feed through breastfeeding. These rates are higher compared to breastfeeding rates for premature babies in the US (62%) and Australia (80–86%) and are similar to the initiation rate of breastfeeding for term babies in Denmark (99%) [123,124]. In a study conducted in six NICUs in Sweden, over 85% of preterm babies were exclusively breastfed, which is a high prevalence compared to other countries. For instance, in Canada, 14% of preterm babies were exclusively breastfed, and 82% were partially breastfed during the 6- to 8-week postpartum period. Furthermore, a higher percentage (55%) of exclusively breastfed infants was observed at 4 months of age in Canada (Calgary) [125,126]. 

The stay of a preterm baby in the NICU is very challenging for breastfeeding promotion due to the separation of the mother from the baby and the difficulties mothers face in initiating and maintaining lactation and establishing breastfeeding. Inadequate milk production leads mothers to experience feelings of failure and frustration. Providing maternal milk allows the mother and her child to be physically and emotionally connected. As a result, the mother engages in caring for her newborn. Receiving mothers together with their babies in the NICU, minimizing pacifier use, and promoting skin-to-skin contact can contribute to earlier establishment of breastfeeding for preterm babies. In these circumstances, the mother can obtain the support needed to fulfil her maternal role [127,128].

## 4. Continuous Monitoring in Neonatal Care and Milk Quality Assessment

Quality monitoring of human milk is important due to compositional changes during storage, and various methods based on analyses of redox capacity and antioxidative activity of this biological fluid are available. The composition of preterm and term milk has been analyzed by various studies. The most significant changes in milk composition occur from colostrum to mature milk. During this period, the protein content decreases, and fat increases, in both term and preterm milk. Therefore, it is essential to carefully determine the energy content in donated term milk that does not meet the energy needs of preterm infants [49,51,129,130]. Thermal treatments, freezing, and pasteurization in milk banks lead to a significant decrease in protein and fat content [131,132,133]. This reduction affects the total energy value of the milk, and therefore, fortification of donor milk is necessary [133,134]. The recent availability of a reliable point-of-care human milk analyzer provides an opportunity to individually target fortification of human milk in the NICU. However, very few NICUs perform individualized fortification due to barriers such as determining recipients, collecting a representative sample, and costs [135]. Comparing the principles and results of common spectroscopic and electrochemical methods, electrochemical voltametric methods are found to be fast, sensitive, and able to be used in clinical practice for determining redox quality, TAC, in milk and infant formulas, in human milk after supplementation with human milk fortifiers, and in milk freshness control, especially in neonatal units. Cyclic voltammetry (CV) and differential pulse voltammetry (DPV) are green methods that do not require the use of chemicals and can directly detect TAC in the original, turbid sample [136,137,138,139]. The electrochemical method, potentiometric titration, can determine the redox activity of bioactive milk components in both the aqueous and fatty phases without prior separation. In addition to the abovementioned methods, the electrochemical method of direct polarography allows specific insight into the quality of infant milk, reflecting the amounts of free thiol groups and protein content, making them useful for monitoring the quality of infant formula and human milk for preterm infants before and after supplementation [138,140]. Electron paramagnetic resonance (EPR) spectroscopy provides specific insight into the quantification of redox activity in infant food. EPR spectroscopy has been offered as a fingerprinting method. It can quickly demonstrate the quality of milk. The application of EPR spectroscopy determines that colostrum has better free radical scavenging capacity than does mature milk. Human milk and infant formula captures hydroxyl radicals produced in the Fenton reaction to produce carbon and ascorbyl radicals. Infant formulas are less capable than human milk of eliminating reactive species [62,141]. 

## 5. Human Milk and Microplastics as Challenging Pollutants

In addition to these quality determinants of milk, maternal nutrition and living conditions also influence the composition of human milk. The challenges of everyday life affect the quality of infant food. Therefore, it is important, alongside nutritional and antioxidative characteristics that are monitored for milk quality estimation, to also consider the impact of various pollutants on the quality of infant food [2,142].

While industrial infant foods are produced under controlled conditions, the presence in foods of microplastics as increasingly common environmental pollutants raises concerns. Microplastics are plastic particles ranging in size from 1 to 1000 µm, and plastic particles smaller than 1 µm are referred to as nanoplastics. They have become ubiquitous environmental contaminants in marine and freshwater systems, soil, air, and food [143,144,145,146,147,148]. Exposure to microplastics can occur even before birth. Recently, it was discovered that microplastics can accumulate and move through the human body and placenta, so infants can be exposed to microplastics even before breastfeeding is initiated [149,150]. However, the current literature does not currently provide compelling evidence for any consistent or clinically relevant health consequences in infants exposed to environmental chemicals through breast milk. Available data strongly suggest that the benefits of breastfeeding outweigh the potential harmful effects of contaminants found in human milk. The Nutrition Committee of the French Pediatric Society strongly supports breastfeeding but also calls for public health activities to reduce overall environmental contamination levels, continue breastfeeding promotion, and support research in this field [21,151,152].

**Figure 1:**
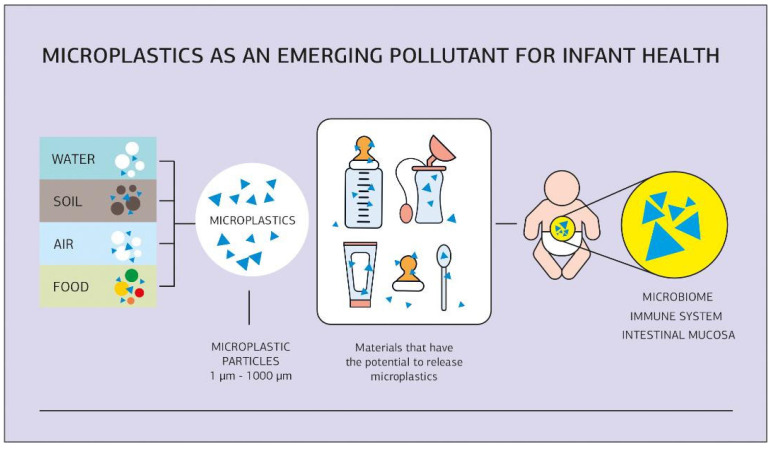
Microplastics as an emerging pollutant for infant health. (o—illustration of matrix particles, Δ—illustration of microplastic particles).

Studies have shown that the use of plastic bottles and pacifiers/nipples in infant feeding and care increases the potential for the ingestion of micro and nanoplastics, which enter the bloodstream and have various potentially harmful effects on the body (Figure 1). In parts of the world with high incomes, including North America and Europe where breastfeeding rates are relatively low, infants could potentially consume from a thousand to over a million microplastic particles daily if infant formula is prepared in polypropylene bottles [153,154]. In China, where glass feeding bottles are more popular, the average exposure of microplastics to infants is estimated to be much lower. Infants in Asia and Africa had the lowest potential exposure [154,155]. Breastfeeding and the use of glass containers for milk storage reduce infants’ exposure to microplastics.

Due to the different characteristics of milk and the ubiquity of microplastics as environmental contaminants, new methods are needed to monitor the quality of infant foods and human milk and the health of pregnant and lactating mothers, to provide a solid foundation for further development and advancement in human milk research. Given the lack of relevant studies, these findings emphasize the need to investigate the contribution of plastic products to microplastic exposure during the lactation period [21,156]. The demonstration in human breast milk of a wide spectrum of potentially harmful chemicals, even if they cannot be classified as health risk factors, should still encourage preventive efforts primarily based on avoidance during pregnancy and lactation. This means paying particular attention to the origin, handling, and storage of food, avoiding/reducing the use of personal care products that often contain endocrine-disrupting chemicals, minimizing indoor pollution, and reducing contact with external pollutants as much as possible [157].

Microplastic particles are present in various sources such as water, soil, air, and food. Different materials used in food packaging and processing have the potential to release microplastics into infant food. This emerging pollutant can have a significant impact on the microbiome and intestinal mucosa, the immune system, and ultimately, the health of infants.

## 6. Conclusions

Human milk, as the primary available biological fluid for infants, is the easiest means to ensure their proper development. Numerous clinical studies from various aspects have shown that breast milk is the best source of nutrition for infants, especially preterm infants. Mother’s milk is the gold standard for infant nutrition, not only due to its nutritional properties but predominantly due to its biological value, stemming from the presence of enzymes, immune-protective substances, hormone-like compounds, and prebiotic oligosaccharides. The benefits of breastfeeding, and particularly the mother’s own milk, for preterm infants are numerous. These benefits for preterm infants include reduced morbidity, shorter treatment duration, and lower incidences of diarrhea, pneumonia, atopic dermatitis, and obesity, and therefore, breastfeeding also has favorable socio-economic aspects. In situations where the mother’s own milk is not available, donor milk or infant formula provides energy and nutritional support for adequate growth and development of her infant. However, the benefits of donor milk are significant, and it is highly preferable to formula. To the best of our current knowledge, the advantages of breastfeeding outweigh the potential harmful effects of microplastics in human milk. However, research is required on this topic, as the impact of microplastics on infant and human health and development cannot be ignored. 

## Data Availability

No new data were created or analyzed in this study. Data sharing is not applicable to this article.
